# Long Non-coding RNAs in Gammaherpesvirus Infections: Their Roles in Tumorigenic Mechanisms

**DOI:** 10.3389/fmicb.2020.604536

**Published:** 2021-01-15

**Authors:** Wen Liu, Yan Zhang, Bing Luo

**Affiliations:** ^1^Department of Pathogenic Biology, School of Basic Medicine, Qingdao University, Qingdao, China; ^2^Department of Clinical Laboratory, Zibo Central Hospital, Zibo, China

**Keywords:** gammaherpesvirus, Epstein–Barr virus, KSHV, lncRNA, tumorigenesis

## Abstract

Long non-coding RNAs (lncRNAs) regulate gene expression at the epigenetic, transcriptional, or posttranscriptional level by interacting with protein, DNA, and RNA. Emerging evidence suggests that various lncRNAs are abnormally expressed and play indispensable roles in virus-triggered cancers. Besides, a growing number of studies have shown that virus-encoded lncRNAs participate in tumorigenesis. However, the functions of most lncRNAs in tumors caused by oncogenic viruses and their underlying mechanisms remain largely unknown. In this review, we summarize current findings regarding lncRNAs involved in cancers caused by Epstein–Barr virus (EBV) and Kaposi’s sarcoma herpesvirus (KSHV). Additionally, we discuss the contribution of lncRNAs to tumor occurrence, development, invasion, and metastasis; the roles of lncRNAs in key signaling pathways and their potential as biomarkers and therapeutic targets for tumor diagnostics and treatment.

## Introduction

Infectious diseases are an important cause of human cancer, of which approximately 15–20% are virus-triggered. The viruses responsible are thus denoted as oncoviruses or oncogenic viruses. Studies of oncogenic viruses have proved invaluable in understanding the mechanisms of cancer occurrence and progression. Several cancer biomarkers have been discovered and have guided the exploitation of new tumor treatment strategies. Human oncogenic viruses include DNA viruses: high-risk human papillomavirus (HPV), Merkel cell polyomavirus (MCPyV), hepatitis B virus (HBV), Epstein–Barr virus (EBV), and Kaposi’s sarcoma-associated herpesvirus (KSHV); RNA viruses: hepatitis C virus (HCV) and human T-cell leukemia virus type 1 (HTLV-1); as well as some serotypes of human adenoviruses, even though they have not been linked to any human cancer (Grassmann et al., 2008; Schafer et al., 2015; Krump and You, 2018). Among them, EBV and KSHV are both large double-stranded DNA (dsDNA) viruses, and they can establish long-term or even lifelong chronic infections that show no obvious post-infection symptoms. These two viruses share many features that can initiate many lymphoproliferative diseases and multitudinous malignancies (Krump and You, 2018). EBV is the first human tumor virus infecting more than 90% of the world’s population. A lifelong latent EBV infection is associated with multiple human cancers, including Burkitt’s lymphoma (BL), Hodgkin’s lymphoma (HL), natural killer (NK)/T cell lymphoma, nasopharyngeal carcinoma (NPC), and some gastric carcinomas (GCs) (Saha et al., 2010; Young et al., 2016). KSHV (also known as human herpesvirus 8), which also belongs to the gammaherpesvirus subfamily, mainly causes Kaposi’s sarcoma (KS) (da Silva and de Oliveira, 2011). KS is the most common AIDS-associated malignancy (Ganem, 2010). However, the underlying mechanisms of these viruses in promoting tumorigenesis and development remain poorly understood.

As large dsDNA gammaherpesviruses, both EBV and KSHV should evade the innate immune response mainly by restricting the expression of viral genes to establish a stable infection in the host (Jangra et al., 2019). EBV and KSHV can alter the expression of viral genes *via* genetic regulation directly, encoding various viral microRNAs (miRNAs), or changing the expression or function of host genes (Lung et al., 2009; Mansouri et al., 2014; Zhang Y. et al., 2018). The expression pattern of EBV viral proteins is disease-specific and is typically identified as four latency patterns: type III, type II, type I, and type 0 latency (Young and Rickinson, 2004). EBV latency proteins, especially Epstein–Barr nuclear antigen 1 (EBNA1) and latent membrane proteins (LMP-1 and LMP-2A), have been well studied and are considered cell-transforming and carcinogenesis-promoting oncoproteins (Shair and Raab-Traub, 2012; Frappier, 2015; Wang L.W. et al., 2017). Recently, EBV non-coding RNAs (ncRNAs), *RPMS1*, EBV miRNAs, and EBER1/2 have gained emphasis. They were demonstrated as significantly affecting oncogenesis by interfering with the cell cycle, host immune responses, and apoptosis (Marquitz et al., 2015; Albanese et al., 2017; Skinner et al., 2017; Zhang J. et al., 2018; Liu et al., 2020). Likewise, KSHV employs oncoproteins and ncRNAs such as latency-associated nuclear antigen (LANA), viral FLICE inhibitory protein (vFLIP), and KSHV-encoded miRNAs to achieve latent infection and tumorigenic processes (Samols et al., 2007; Ballon et al., 2011; Yang et al., 2014; Wei et al., 2016; Hussein et al., 2019). Thus, the use of ncRNAs to regulate the biological process of host cells is an important strategy in virus infection to avoid eliciting immune clearance by the host (Ungerleider et al., 2018).

Only a very small part of the mammalian genome encodes protein-coding genes, with most of the genome transcribed as ncRNAs (Holoch and Moazed, 2015). With the development of sequencing technology and bioinformatics in the past two decades, we have a deeper understanding of the ncRNA richness and their possible roles in human cells – we now realize that these non-coding transcripts are not transcriptional noise, but serve important biological functions. NcRNAs are roughly divided into three types: miRNAs, long non-coding RNAs (lncRNAs), and circular RNAs (circRNAs) (Chen et al., 2016). Current studies have revealed multiple functions of lncRNAs in various cell processes by regulating gene expression, including cell migration, proliferation, cell cycle, apoptosis, and autophagy (Kung et al., 2013; Huarte, 2015). The abnormal expression of lncRNAs, which involves various important pathways, is closely related to the occurrence and development of tumors (Huarte, 2015; Bullard et al., 2018; Castro-Oropeza et al., 2018; Lin, 2020). LncRNAs encoded by oncogenic viruses have been considered important cofactors that participate in tumorigenesis (Zhang et al., 2016; Liu and Ding, 2017). Due to their close relationship with tumor initiation, invasion, and metastasis, and drug sensitivity and resistance, these lncRNAs can serve as novel therapeutic targets and treatment tools for viral tumors. Recent studies have attempted to clarify the molecular mechanism of these RNAs, thereby providing new insight into their functions and applications (Huarte and Rinn, 2010). Here, we focus on the recent understanding of lncRNAs involved in EBV- and KSHV-associated tumors, especially on their roles in the establishment of latent infection and oncogenesis.

## The Molecular Function of Long Non-Coding RNAs

As important regulatory RNAs in tumor biology, lncRNAs are involved in multiple major biological functions, for instance, cell proliferation, invasion, metastasis, and apoptosis (Lin, 2020; Zhou et al., 2020). Based on their genomic location, lncRNAs are mainly categorized into five types: antisense, bidirectional, intronic, enhancer-associated, and intergenic (Robinson et al., 2020). Mechanistically, lncRNAs function at all epigenetic, transcriptional, and posttranscriptional levels with diverse mechanisms. LncRNAs regulate histone modification and DNA methylation in pre-transcriptional regulation (Schmitz et al., 2016). By directly binding to transcription factors or chromatin modifiers, lncRNAs act as a scaffold to direct them to precise locations in the genome by *cis* or *trans* regulation (Wilusz et al., 2009). Besides, lncRNAs regulate mRNA transcripts by affecting the stability, changing the splicing activity, editing modifications, or regulating RNA subcellular localization. It can act as a competitive endogenous RNA (ceRNA) or “sponge” for miRNAs, indirectly de-repressing the expression of the mRNA that would be targeted by these miRNAs (Huarte, 2015; Li T. et al., 2016; Yan et al., 2019; Robinson et al., 2020). Furthermore, cellular lncRNAs can also be targeted by miRNAs (Sethuraman et al., 2018). However, further evidence with detailed regulatory mechanism is required to support this view.

## Viral-Encoded Long Non-Coding RNAs of Epstein–Barr Virus and Kaposi’s Sarcoma Herpesvirus

Viral-encoded lncRNAs are expected to play an important role in maintaining stable virus infection and promoting tumorigenesis and tumor progression (Zheng, 2010; Li Z. et al., 2016). Currently, a small number of known EBV- and KSHV-encoded lncRNAs exists, and their biological significance remains to be determined. However, with the advancement of technology and more in-depth analysis of the viral genome, we have gradually discovered these virus-related ncRNAs and began studying their functions in viral-associated malignancies. EBV- and KSHV-encoded viral genes are mostly associated with establishing stable latent infections, while lncRNAs, as important ncRNAs, are certainly involved in latency regulation. Here, we describe several EBV- and KSHV-encoded lncRNAs that are better-characterized.

### Epstein–Barr Virus-Encoded Long Non-coding RNAs

Epstein–Barr virus miRNAs and lncRNAs can target each other and share several common signaling pathways, forming an interconnected, complex molecular regulatory network. EBV also encodes its own viral lncRNAs such as EBV bamHI-A rightward transcripts (BARTs) expressed in all EBV-associated diseases. BARTs contain several forms of splicing in EBV-infected cells. A study detected *BARF0*, *RPMS1*, *RPMS1A*, and *A73* genes as four major splicing forms encoded by BART, but no endogenous BART-translated protein was detected (Yamamoto and Iwatsuki, 2012). Full-length BART RNA reportedly functions as lncRNA in the nucleus of GC cells, which induces transcriptional reprogramming associated with latent EBV infection. In the report, eight genes were strongly downregulated by BART lncRNA, including *RASIP1*, *SLC7A11*, *PGC*, *CDH11*, *RNF144B*, *ATF5*, *VEGFA*, and *ITGA6* (Marquitz et al., 2015). NPC is the most common malignant tumor of head and neck squamous cell carcinoma. It occurs in southern China and Southeast Asia and is closely related to EBV infection. More than 90% of non-differentiated NPC cases are EBV-positive. High levels of BART expression have also been reported in NPC. The regulation of BARTs is postulated to be related to EBV pathogenesis in NPC (Verhoeven et al., 2016), and BART lncRNAs are involved in the epigenetic regulation of host gene expression in NPC. BART lncRNA is located in the nucleus and regulates the expression of interferon beta 1 (*IFNB1*) and chemokine (C-X-C motif) ligand 8 (*CXCR8*) by inhibiting polymerase II (Pol II) transcription (Verhoeven et al., 2019). Together, BART lncRNAs participate in transcription reprogramming of host gene expression in both Epstein–Barr virus-associated gastric carcinoma (EBVaGC) and NPC. Nevertheless, most of the target genes that interact with BART lncRNAs remain unknown.

Epstein–Barr virus-encoded *BHLF1* is an early lytic gene that is expressed at a low level during the initiation of latency but at a high level during reactivation. *BHLF1* encodes several RNAs and performs non-coding functions (lncRNA and circRNA) during the viral replicative cycle (Ungerleider et al., 2019; Yetming et al., 2020). BHLF1 lncRNA acts as the essential promoter of the origin of lytic replication (OriLyt) to promote DNA replication. Efficient protein expression of BHLF1 requires the EBV posttranscriptional regulator protein SM, which is expressed only in the replicative cycle. Therefore, BHLF1 lncRNA may have an important role in the lytic cycle. Meanwhile, Yetming et al. (2020) indicated that BHLF1 lncRNA also contributes to viral latency.

### Kaposi’s Sarcoma Herpesvirus-Encoded Long Non-coding RNAs

Kaposi’s sarcoma herpesvirus is closely associated with the occurrence of various malignant tumors. Among them, KS is a common tumor in patients with AIDS, which causes death (Chang et al., 1994). KSHV is a dsDNA virus consisting of ∼165-kb genome that encodes approximately 90 viral proteins and many ncRNAs (Campbell et al., 2020). To prevent elimination by the host immune response, KSHV establishes a latent infection. During latency, KSHV restricts viral gene expression to a few latent proteins and an array of viral microRNAs and lncRNAs (Samols et al., 2007; Schifano et al., 2017). Three main latency genes are commonly expressed in KS and PEL: *LANA*, *vFLIP*, and the viral homolog for a cellular D cyclin (*vCyc*). As summarized by Hussein et al. (2019), the KSHV genome also encodes 25 mature miRNAs.

**FIGURE 1:**
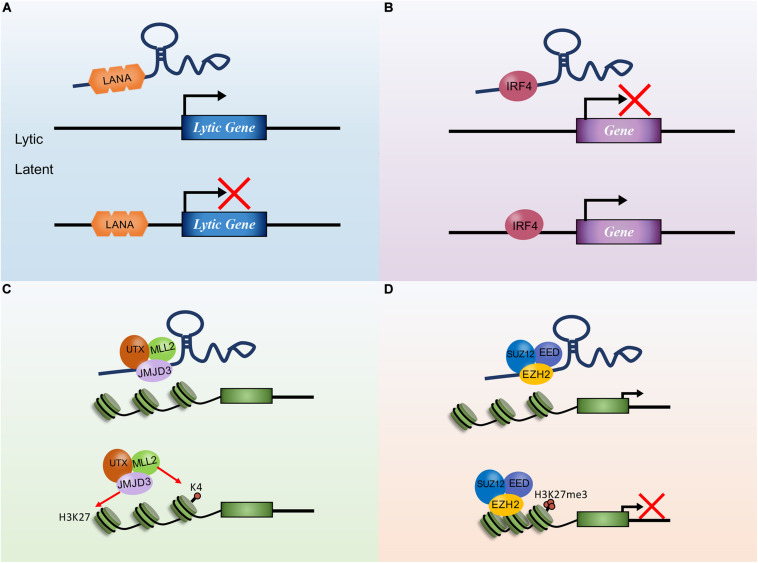
The mechanisms of Kaposi’s sarcoma-associated herpesvirus (KSHV) polyadenylated nuclear RNA (PAN RNA) action. **(A)** PAN RNA directly interacts with IRF4 transcription factor and represses IRF4-driven transcription. **(B)** PAN RNA sequesters KSHV latency-associated nuclear antigen (LANA) protein from the KSHV genome to promote KSHV lytic replication. **(C)** PAN RNA binds to EZH2 and interacts with PRC2 to promote gene expression by repressing H3K27 methylation. **(D)** PAN RNA interaction with the histone demethylases UTX and JMJD3 to regulate gene expression by removing repressive H3K27Me.

Approximately 16 potential KSHV lncRNAs have been reported as present in infected cells (Schifano et al., 2017). To date, polyadenylated nuclear RNA (PAN RNA) is the most important and well-characterized KSHV lncRNA, which was first described in 1996 (Sun et al., 1996; Zhong et al., 1996). PAN RNA is a multifunctional regulatory transcript and plays important roles in reactivation and replication, viral gene expression, and immune modulation by interacting with viral and cellular proteins and DNA (Figure 1; Borah et al., 2011; Rossetto and Pari, 2011, 2014; Rossetto et al., 2013; Campbell et al., 2014b; Sztuba-Solinska et al., 2017). PAN RNA is abundantly expressed throughout lytic replication and plays a vital role in viral replication. Besides interacting with cellular proteins, it can also interact with viral proteins (Rossetto and Pari, 2014). The significant and diverse functions of PAN RNA in KSHV lytic replication have been well described (Campbell and Izumiya, 2020).

Polyadenylated nuclear RNA has been implicated in transcription and chromatin remodeling, and LANA is a nuclear protein expressed during latent KSHV infection that interacts with PAN RNA (Song et al., 2001). By interacting with PAN RNA, LANA dissociates from the viral episomes, facilitating lytic reactivation (Campbell et al., 2014a). Recent reports have shown that PAN RNA plays significant roles such as binding to the transcription factor IRF4 to inhibit transcription of downstream genes, interacting with the lysine demethylase JMJD3 complex to regulate gene expression by removing repressive H3K27Me, and binding to the lysine methylase EZH2 [a histone methyltransferase subunit of the epigenetic regulator polycomb repressor complex 2, PRC2, which suppresses gene expression by adding three methyl groups to lysine 27 of histone 3 (H3K27)] to regulate gene expression *via* repressing methylation (Rossetto and Pari, 2012, 2014; Kim and Roberts, 2016). KSHV can also induce EZH2 expression through the expression of vFLIP and LANA to promote angiogenesis (He et al., 2012). Additionally, PAN RNA was shown to interact with histones H1 and H2A, PRC2, and KSHV open reading frames (ORFs) (26, 57, and 59). These studies suggest that PAN RNA potentially affects the expression of KSHV and cellular genes and functions in epigenetic gene regulation, similar to cellular lncRNAs.

Antisense-to-latency transcript (ALT) is a potential non-coding transcript antisense that was discovered from genome-wide tiled microarray studies. However, its role in KSHV latency and pathogenesis remains unknown (Arias et al., 2014; Schifano et al., 2017). As a potential KSHV lncRNA, the function of ALT during infection and pathogenesis remains unknown (Bullard et al., 2018). In the future, further research should be conducted to clarify the roles and molecular mechanisms of viral lncRNAs in oncogenesis and its clinical applications.

## Cellular Long Non-Coding RNAs Regulated by Epstein–Barr Virus and Kaposi’s Sarcoma Herpesvirus

Viral infections are reported to regulate host lncRNAs and play an important role in the occurrence and progression of viral-related tumors. EBV and KSHV deregulate host lncRNAs *via* direct interaction through using latency-associated proteins or through viral miRNAs to drive tumorigenesis (Table 1).

**TABLE 1:** Cellular lncRNAs regulated by EBV and KSHV.

Viral genes	LncRNAs	Functions	References
EBV miR-BART14	AFG3L1P	Mitochondrial fusion; apoptosis	Li C.W. et al., 2018
EBV miR-BART6-3p	LOC553103	EMT; migration and invasion	He et al., 2016; Wang et al., 2020
EBV	7SL	Cell cycle; EBV tumorigenesis	Gallo et al., 2017
	H19		
	H19 antisense		
	p53 mRNA		
EBV BHLF1, LF3, BHRF1, BNLF2a	SNHG8	Cell cycle; DNA repair; EMT and ribosomal function; cell proliferation and colony formation	Huang et al., 2016; Lin et al., 2018; Naseem et al., 2018
KSHV miR-K12-11	MIR17HG, MIR155HG, AFAP1-AS1	TGF-β signaling	Sethuraman et al., 2018
KSHV miR-K12-9	H19	Tumor initiation; progression and metastasis	Sethuraman et al., 2018
KSHV miR-K12-6-3p	UCA1	Proliferation; migration	Zuo et al., 2017; Sethuraman et al., 2018
KSHV miR-K12-10b, 7	TUG1	Binding to PRC2 complexes	Sethuraman et al., 2018
KSHV miR-K12-3, 4-3p, 8	GAS5	Tumor suppressor	Sethuraman et al., 2018
KSHV miR-K12-4-3p, 7, 8, 9, 10a, 10b, 11, 12 EBV	MALAT1	Proliferation and metastasis	He et al., 2017; Sethuraman et al., 2018
KSHV miR-K12-4-3p	DLEU2	Inducing apoptosis; histone modifications, DNA methylation	Sethuraman et al., 2018; Chi et al., 2019
KSHV vIRF1	lnc-OIP5-AS1	Proliferation, migration, and invasion	Li W. et al., 2019

*BARTs, bamHI-A rightward transcripts; EBV, Epstein–Barr virus; EMT, epithelial–mesenchymal transition; KSHV, Kaposi’s sarcoma-associated herpesvirus; lncRNA, long non-coding RNA; MALAT1, metastasis-related lung adenocarcinoma transcript 1; TGF-β, transforming growth factor-β.*

Epstein–Barr virus encodes two clusters including 44 mature miRNAs, many of which promote cancer progression by targeting host genes. However, few EBV miRNAs showed inhibition in tumor cell migration and invasion. EBV viral miR-BART14 was found to repress the expression of lncRNA AFG3L1P, which is associated with mitochondrial fusion and may trigger apoptosis *via* bioinformatics identification (Li C.W. et al., 2018). LOC553103 has been reported to promote epithelial–mesenchymal transition (EMT) and thereby increase the invasive and metastatic capability of NPC cells. EBV-encoded miR-BART6-3p directly targets and downregulates lncRNA LOC553103 to inhibit NPC and GC cell invasion and metastasis *via* the regulation of EMT-related molecular targets, such as upregulating E-cadherin and downregulating β-catenin, Snail, and N-cadherin (He et al., 2016). Besides, EBV regulates cell cycle and inhibits cell proliferation *via* the miR-BART6-3p/LOC553103/STMN1 axis (Wang et al., 2020).

Gallo et al. (2017) analyzed the expression of 90 lncRNAs in human EBV-transformed lymphoblastoid cell line (LCL) and peripheral blood mononuclear cells (PBMCs) and found that four lncRNAs showed higher expression in the LCL, including 7SL, H19, H19 antisense, and p53 mRNA. H19 and H19 antisense were enriched in LCL exosome cargo (Gallo et al., 2017) and may contribute to EBV-driven tumorigenesis. Reports indicate that NPC cells are ubiquitously infected with EBV. Li X.X. et al. (2018) found that 62 genes *trans-*regulated by lncRNAs were involved in the EBV infection pathway in NPC. Moreover, they identified eight lncRNAs dysregulated in NPC (C666-1) and GC (AGS-EBV) cells, including lncRNA-BC200, metastasis-related lung adenocarcinoma transcript 1 (MALAT1), LINC00672, LINC00982, IGFBP7-AS1, LOC100128494, LINC02067, and LOC100505716 (Li X.X. et al., 2018). However, little is known about the EBV–lncRNA interactions in NPC.

Small nucleolar RNA host gene 8 (SNHG8) is a recently reported lncRNA that exhibits abnormal expression patterns and was significantly associated with shorter survival times in GC (Huang et al., 2016; Lin et al., 2018). SNHG8 has been identified as an EBVaGC-specific expression lncRNA and is indicated to affect several GC-specific pathways. The hypergeometric statistical test showed that SNHG8 interacts significantly with EBV proteins BHLF1, LF3, BHRF1, and BNLF2a and targets DNA repair-related molecules, EMT, and cancer progression, such as TRIM28, NAP1L1, and TRPM7 (Huang et al., 2016). *In vitro* and *in vivo* studies have proved that SNHG8 promotes cell proliferation by acting as a proto-oncogene and promoting cell proliferation and participating in gastric carcinogenesis (Chen et al., 2017; Liu et al., 2018).

High expressions of MALAT1, AFAP1-AS1, and AL359062 were considered novel serum biomarkers for the diagnosis and prognosis of NPC, which have been demonstrated to exhibit a close association with the stage of lymph node metastasis and EBV infection (He et al., 2017). Nevertheless, there is still a lack of effective verification.

Kaposi’s sarcoma herpesvirus can produce miRNA at different stages of infection, which can promote the virus’s malignant transformation of host cells. Many recent studies have demonstrated that host cell lncRNAs are targeted by KSHV-encoded miRNAs. Computational analysis identified and cataloged thousands of putative lncRNA targets of gammaherpesvirus miRNAs and found that 99 lncRNAs in the putative viral miRNA targets are cancer-associated. Further analysis revealed that GAS5, DLEU2, and nuclear paraspeckle assembly transcript 1 (NEAT1) were previously identified in lymphoma. In addition, MIR17HG, MIR155HG, MALAT1, and AFAP1-AS1 were the potential targets of KSHV-encoded miR-K12-11 (Sethuraman et al., 2018). Thirty-five cancer-relevant lncRNAs were identified, including the oncogenic lncRNAs MALAT1 and UCA1 and lncRNAs GAS5 and TUG1, which act as tumor suppressors (Sethuraman et al., 2018). Another study identified 126 lncRNAs including MEG3, ANRIL, and UCA1, which are closely implicated in cancer as putative targets of KSHV miRNA. They were found to reside in the nucleus and interact with miRNAs in endothelial cells (Sethuraman et al., 2017). Together, the lncRNAs that may interact with KSHV miRNAs in cancers are GAS5, MIR17HG, DLEU-2, MIR155HG, MALAT1, AFAP1-AS1, UCA1, TUG1, MEG3, ANRIL, and H19.

Several lncRNAs are abnormally expressed in KSHV-infected cells. LncRNA MIR17HG is the host gene of the miR-17-92 cluster, which is upregulated during KSHV infection, resulting in downregulation of transforming growth factor (TGF)-β signaling. UCA1 deregulation by KSHV increased the proliferation and migration of endothelial cells. Upregulation of UCA1 by TGF-β also promotes the development and progression of GC by augmenting GC cell proliferation and invasive and migratory capabilities (Zuo et al., 2017). MEG3 is downregulated in a variety of malignant tumors and acts as a tumor suppressor. LINC00313, which is upregulated by KSHV reactivation, was shown to interact with HIV Tat (Yang et al., 2020).

Kaposi’s sarcoma herpesvirus-encoded oncogene viral interferon regulatory factor 1 (*vIRF1*) hijacks the lncRNA OIP5 antisense RNA 1 (lnc-OIP5-AS1)/miR-218-5p axis to regulate the high-mobility group box 2 (*HMGB2*) and cytidine/uridine monophosphate kinase 1 (*CMPK1*) and promotes endothelial cell migration, invasion, and proliferation (Li W. et al., 2019). In addition, lnc-OIP5-AS1 increased DNA methylation of the pre-miR-218-1 promoter, inhibiting miR-218-5p expression. Lnc-OIP5-AS1 also exerts its oncogenic functions and is consistently upregulated in GC cells (Rossetto and Pari, 2011).

Metastasis-related lung adenocarcinoma transcript 1 is a well-known lncRNA associated with several human cancers. It is closely related to tumor cell proliferation, angiogenesis, migration, invasion, and apoptosis and is regulated by various factors (Wang et al., 2014, 2018a; Lee et al., 2017; Du et al., 2018; Zhao et al., 2018; Duan et al., 2019). High expression of MALAT1 can be detected in the serum of GC patients, suggesting that MALAT1 is a potential biomarker for the diagnosis. MALAT1 can also be used as a therapeutic target of specific tumors (Hua et al., 2016). MALAT1 promotes cell proliferation and inhibits apoptosis of GC cells. By directly binding to *SOX2* mRNA and enhancing the stability of *SOX2* mRNA, MALAT1 can increase the stemness of GC cells (Xiao et al., 2019). Moreover, MALAT1 regulates the IL-21R signaling pathway in GC cells by competitively binding miR-125a. IL-21R has been identified as an oncogenic gene that promotes cell proliferation and invasion (Yan et al., 2019). Evidence suggests that the overexpression of MALAT1 increased cell proliferation, invasion, and migration in GC possibly through activation of the phosphoinositide 3-kinase/protein kinase B (PI3K/AKT) pathway (Zhu et al., 2019). Specifically, MALAT1 overexpression promotes protein phosphorylation of PI3K, AKT, and signal transducer and activator of transcription 3 (STAT3) in GC cells (Dai et al., 2020). In addition, MALAT1 was demonstrated to competitively bind miR-181a-5p with *AKT3*, upregulating the AKT3 protein level to enhance cell growth (Lu et al., 2019). Furthermore, the MALAT1/miR-183/sirtuin 1 (SIRT1) axis was found to regulate cell apoptosis and autophagy *via* the PI3K/AKT/mammalian target of rapamycin (mTOR) pathway in GC (Li H. et al., 2019). MALAT1 promotes chemotherapy resistance of GC cells by acting as a miRNA sponge. It also inhibits miR-30b expression by direct interaction and increases the expression of *ATG5* by competitively binding miR-30b in GC, thereby potentiating autophagy-related cisplatin resistance (Xi et al., 2019); promotes GC cell oxaliplatin resistance *via* modulation of ZFP91 by sponging miR-22-3p (Zhang et al., 2020); promotes the invasion and metastasis of GC by regulating EGFL7 expression (Deng et al., 2016); and promotes GC tumorigenesis and progression *via* the MALAT1/miR-1297/*HMGB2* axis or by facilitating vasculogenic mimicry (VM) and angiogenesis through multiple related signaling pathways (Li J. et al., 2017; Li Y. et al., 2017).

**FIGURE 2:**
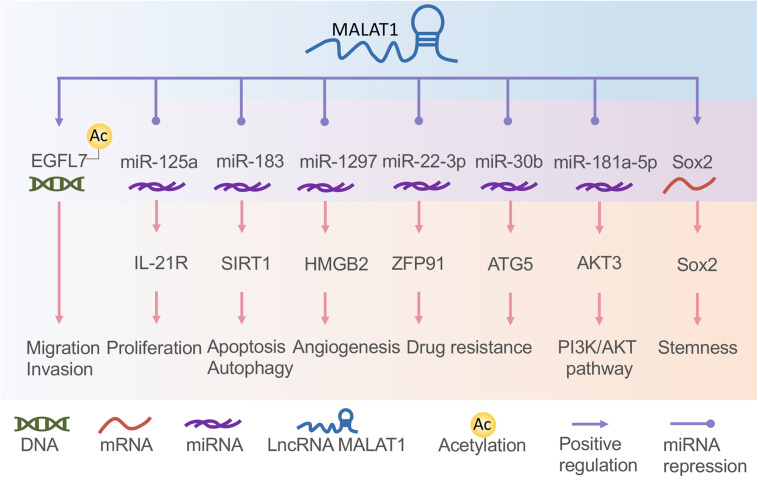
The functions of long non-coding RNA (lncRNA) metastasis-related lung adenocarcinoma transcript 1 (MALAT1) in gastric cancer and nasopharyngeal carcinoma. MALAT1 interacts with DNA, messenger RNA (mRNA), and microRNA (miRNA) to regulate tumor cell proliferation, migration, invasion, angiogenesis, drug resistance, apoptosis, and autophagy.

In NPC cells, MALAT1 promotes invasion and EMT *via* the de-repression of Capn4 by sponging miR-124 (Shi et al., 2017). The excessive expression of MALAT1 also downregulates E-cadherin, while upregulating N-cadherin and vimentin (Lee et al., 2017). Interestingly, MALAT1 can be influenced by miR-124 and miR-25 in NPC. For instance, TGF-β increases MALAT1 expression by repressing miRNA-124 (Du et al., 2018). MALAT1 is negatively regulated by miR-25 for Ago2-dependent degradation (Hua et al., 2016). These findings imply that MALAT1 may function crucially in all tumor processes induced by gammaherpesvirus. The functions of lncRNA MALAT1 in gastric cancer and NPC are summarized in Figure 2.

## The Roles of Long Non-Coding RNAs in Epstein–Barr Virus-Associated Gastric Carcinoma

Gastric cancer is one of the most frequently occurring cancers worldwide. EBVaGC accounts for approximately 9% of the total number of GCs (Li et al., 2010; Iizasa et al., 2012; Nishikawa et al., 2014; Chen et al., 2015; Bae and Kim, 2016; Ribeiro et al., 2017; Castaneda et al., 2019; Qiao et al., 2019) and is one of the four major molecular subtypes (Cancer Genome Atlas Research Network, 2014). EBVaGC has a unique clinicopathological characteristic in that the frequency of lymph node metastasis is significantly lower (van Beek et al., 2004). However, the molecular mechanism of EBVaGC remains elusive (Tsao et al., 2017). Usually, EBV performs type I latency in EBVaGC, expressing EBNA1, EBERs, and BARTs. LMP2A is expressed in approximately 50% of EBVaGC cases (Naseem et al., 2018). Therefore, miRNAs and lncRNAs play important regulatory roles in EBVaGC. Jing et al. (2018) established a complex regulatory network of transcription factors, lncRNAs, and EBV-related miRNAs in EBVaGC using multilevel expression data and a bioinformatics approach. They analyzed the gene expression profiling data sets (GSE51575) and built a transcription factor regulation network using Cytoscape. By establishing the ceRNA network, they found that lncRNA RP5-1039K5.19 and TP73-AS1 may participate in the gene regulation of EBVaGC (Jing et al., 2018).

## Long Non-Coding RNAs in Exosomes

Extracellular vesicles are mainly divided into apoptotic bodies, microvesicles, and exosomes. Exosomes are widely involved in material transportation and information transmission between cells. DNA, RNA, proteins, and other molecules are carried by exosomes and released from tumor cells, allowing the exchange of information and regulating tumor formation, growth, angiogenesis, metastasis, and drug resistance (Liu et al., 2017). In recent years, tumor-associated substances in exosomes have been a research hotspot, in particular, ncRNAs. New cancer-related diagnostics and prognostics based on ncRNA are developing rapidly. Recent studies have shown that lncRNAs can be secreted into peripheral blood by microvesicles and exosomes to affect cell–cell interactions (Liu et al., 2017; Xie et al., 2019). Thus, exosomal lncRNAs may become novel early diagnostic biomarkers for cancer progression. Herpesvirus-associated biomolecules can be shuttled from host cells to recipient cells to achieve an infection and even oncogenesis. Several studies have demonstrated the functions and roles of exosomes in the EBV tumorigenic process (Canitano et al., 2013; Aga et al., 2014; Teow et al., 2017). LncRNA ZFAS1 has been found to be elevated in serum exosomes of GC patients, indicating that it plays an active role in GC progression and represents a biomarker for GC diagnosis (Xie et al., 2019). However, no research has found that lncRNAs exist in KSHV-associated neoplasma-derived exosomes, whether they are of virus or cell origin (Zheng et al., 2019).

## Potential Long Non-Coding RNAs Regulated by Epstein–Barr Virus and Kaposi’s Sarcoma Herpesvirus

### H19

**FIGURE 3:**
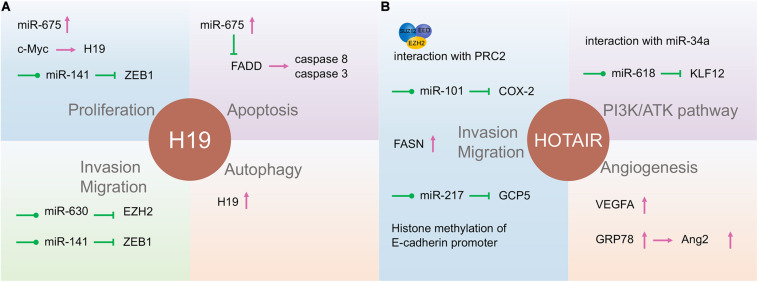
The function of long non-coding RNAs (lncRNAs) H19 and HOTAIR in gastric cancer and nasopharyngeal carcinoma. H19 **(A)** and HOTAIR **(B)** are involved in almost all tumor processes, and HOTAIR is more closely associated with tumor progression. They participate in the tumor processes through direct action, adsorption of microRNA (miRNA), or some currently unknown way to regulate gene expression.

Long non-coding RNA H19, one of the best-known imprinted genes, has been widely proven to have carcinogenic effects (Matouk et al., 2007; Raveh et al., 2015). H19 is highly expressed and correlated with proliferation, invasion, and migration in most types of cancers and acts as a potential diagnostic and prognostic target (Figure 3; Zhang et al., 2014; Yoshimura et al., 2018; Ghafouri-Fard et al., 2020). H19 and miR-675 were upregulated in GC cells and tissues, promoting cell proliferation and inhibiting cell apoptosis. Further studies indicated that the H19/miR-675 axis inhibited the expression of Fas-associated protein with a novel death domain (FADD), subsequently inhibiting cleavage cascades of caspase 8 and caspase 3 (Yan et al., 2017). In another study, the expression of lncRNA H19 in GC was induced by c-Myc (Zhang et al., 2014). H19 knockdown studies verified that the pathway most affected by H19 is the EMT process (Zhang et al., 2017). H19 also plays a fundamental role in the regulation of autophagy through the induction of the PI3K/Akt/mTOR pathway in human cancers (Ghafouri-Fard et al., 2020). It has also been reported to have high levels of circulating H19 in GC patients, being inversely related to tumor size. However, the plasma level of H19 decreased significantly after tumor resection (Yörüker et al., 2018). Li X. et al. (2016) verified that H19 is involved in NPC metastasis by upregulating EZH2 expression *via* interaction with miR-630. The association between KSHV and H19 has been discussed above. Nonetheless, the role of H19 in EBV- and KSHV-induced tumors is an extensive subject.

### Nuclear Paraspeckle Assembly Transcript 1

Nuclear paraspeckle assembly transcript 1 is highly expressed as an oncogenic gene in several types of solid tumors (Yu et al., 2017). Lu et al. (2016) reported that NEAT1 increased ZEB1 expression by targeting miR-204. Therefore, high levels of NEAT1 in NPC induce an EMT phenotype and are associated with a poor prognosis (Lu et al., 2016). Furthermore, NEAT1 was substantiated to directly interact with miR-124, promoting tumorigenesis and progression of NPC. NEAT1 upregulation promotes NPC tumorigenesis and progression by regulating the NF-κB signaling pathway (Cheng and Guo, 2017). In gastric cancer cells, upregulation of NEAT1 may promote cell proliferation, migration, and invasion *via* the miR-335-5p/ROCK1 axis (Wang et al., 2018b). NEAT1 was also found to be a target of KSHV miRNAs (Sethuraman et al., 2018).

### HOTAIR

The lncRNA HOX transcript antisense intergenic RNA (HOTAIR), acknowledged as an oncogenic factor in various malignancies, is closely related to the regulation of EMT, including NPC and GC (Figure 3; Nie et al., 2013). HOTAIR mediates the invasion and metastasis of cancer cells *via* interaction with PRC2, which alters the methylation of H3K27 (Gupta et al., 2010). Its expression contributes to NPC tumorigenesis and progression by the upregulation of cyclooxygenase-2 (COX-2) *via* miR-101 sponging and fatty acid synthase (FASN) (Ma et al., 2017; Hu et al., 2018), and the expression is positively correlated with NPC poor prognosis. Besides, HOTAIR promotes angiogenesis in NPC by directly activating vascular endothelial growth factor A (VEGFA) or upregulating VEGFA and Ang2 expression through GRP78 (Fu et al., 2016).

HOX transcript antisense intergenic RNA can also promote the development of GC in various ways. Increased HOTAIR has been associated with lymph node metastasis and clinical stage and positively correlated with poor prognosis in GC patients (Da et al., 2017). This lncRNA participates in the development of GC by promoting GCP5 expression *via* sponging miR-217 (Dong et al., 2019). E-cadherin transcription was inhibited by HOTAIR through histone methylation at the E-cadherin promoter of HOTAIR in GC (Song et al., 2019). HOTAIR can also affect GC cell cycle distribution by regulating P21 and P53 proteins (Xu et al., 2019). HOTAIR could act as a ceRNA to repress miR-618 and subsequently increase KLF12 expression, inhibiting GC progression (Xun et al., 2019). By interacting with miR-34a, HOTAIR may also be involved in the PI3K/Akt and Wnt/β-catenin signaling pathways (Cheng et al., 2018). Thus, lncRNA HOTAIR might be a potentially useful independent prognostic biomarker for GC.

### Other Long Non-coding RNAs

In NPC, the SNHG1 of lncRNA was shown to upregulate an AMPK-related kinase, NUAK1, by inhibiting miR-145-5p and subsequently promoting the migration of NPC cells partly through AKT signaling and EMT (Lan and Liu, 2019).

Small nucleolar RNA host gene 7 has been explored for its potential role in the occurrence and progress of many human malignancies. Its high expression is related to the poor prognosis of patients. SNHG7 is highly expressed in GC tissues and cells and promotes the proliferation of gastric cancer cells and inhibits apoptosis (Wang M.W. et al., 2017).

Long non-coding RNA-ATB was overexpressed in gastric cancer tissues and cells, promoting cell proliferation and invasion through the miR-200s/ZEB axis (Saito et al., 2015; Chen et al., 2018). In addition, it can also promote cell proliferation by regulating miR-141-3p/TGF-β2 axis (Lei et al., 2017).

LINC00312 (also called NAG7) was downregulated in NPC. It was negatively correlated with EBER-1 expression. The low expression of LINC00312 is consistent with its effect of inhibiting cell proliferation and inducing apoptosis. However, it can also stimulate NPC cell invasion (Tan et al., 2002). Interestingly, LINC00312 is negatively correlated with tumor size but positively correlated with lymph node metastasis. LINC00312 activates the JNK2/AP-1/MMP1 pathway and the upstream H-Ras/p-c-Raf pathway and inhibits the expression of estrogen receptor α (ERα), which all play roles in the promotion of NPC invasion and migration (Huang et al., 2009). Therefore, LINC00312 may be an important molecular marker involved in the progression of NPC and affecting metastasis and prognosis. However, the exact mechanism requires further investigation.

LINC0086 is an lncRNA found to be decreased in the serum and tissues of NPC patients. It is associated with tumor stage and lymph node metastasis. Guo et al. (2017) demonstrated that LINC0086 acts as a tumor suppressor by directly targeting miR-214, which plays a carcinogenic role in NPC. Since C666-1 is an EBV-positive NPC cell line, we may observe whether LINC0086 is correlated with EBV through further experiments.

These lncRNAs demonstrate a close relationship with every aspect of GC and NPC initiation and development, thus they may be inextricably linked to EBV. It would be interesting to further characterize their roles in viral-related tumors.

## Conclusion

Viral infection is associated with approximately 20% of all human cancers. In this regard, EBV and KSHV are the two main contributors to the development of various cancers. They establish a lifelong latent infection in host cells through interaction with the host microenvironment. Moreover, to avoid host immune surveillance during latency, only a small part of the viral genes is expressed, including several viral proteins and ncRNAs. Therefore, ncRNAs have great potential for the carcinogenicity of gammaherpesvirus and function at every stage of tumor development.

Emerging evidence has revealed the strong regulatory functions of lncRNAs in cancers. Recent studies have proved the important role of lncRNAs in viral infections and human cancer. However, compared with viral proteins, research on ncRNAs, especially lncRNAs, remains in its infancy. Little is known about the viral lncRNA and virus-related host lncRNAs. Few lncRNAs related to oncogenic viruses have been screened out by bioinformatics methods, but to date, there is no functional verification. Nevertheless, we can gain inspiration from a few well-studied lncRNAs. We highlighted the importance of lncRNAs in the tumorigenicity of gamma herpesviruses. This review summarizes the virus-encoded lncRNAs related to tumors caused by EBV and KSHV, the molecular mechanism of the effect, and host lncRNAs affected by the virus infection. Specifically, the recent discovery of disease-specific lncRNAs in exosomes and peripheral blood could provide theoretical support for the development of more convenient and effective early diagnosis and treatment strategies for cancer. These small molecules stably exist in the blood and are expected to become biomarkers for accurate and sensitive early screening of human cancers. As only a small number of these lncRNAs have been identified, it seems that further study will clarify more tumor-related lncRNAs. LncRNA research is like a mine with rich connotations, waiting for deeper exploitation. The study of viral lncRNAs and the complex regulatory network between viral miRNAs and host lncRNAs will undoubtedly provide a new direction for current cancer research.

## Author Contributions

WL and YZ collected the related papers and wrote the manuscript. BL revised the manuscript. All authors read and approved the final manuscript.

## Conflict of Interest

The authors declare that the research was conducted in the absence of any commercial or financial relationships that could be construed as a potential conflict of interest.

## Abbreviations

EBV: Epstein–Barr virus
KSHV: Kaposi’s sarcoma-associated herpesvirus
NPC: nasopharyngeal carcinoma
GC: gastric carcinoma
KS: Kaposi’s sarcoma
PEL: primary effusion lymphoma
MCD: multicentric Castleman’s disease
LMP: latent membrane protein
EBNA: Epstein–Barr nuclear antigen
LANA: latency-associated nuclear antigen
vFLIP: viral homolog for the cellular FAS-associated death domain-like interleukin-1b converting enzyme (FLICE)-like inhibitory protein
ceRNA: competitive endogenous RNA
BARTs: bamHI-A rightward transcripts
Pol II: RNA polymerase II
vCyc: viral homolog for a cellular D cyclin
PAN RNA: polyadenylated nuclear RNA
PRC2: polycomb repressor complex 2
ALT: antisense-to-latency transcript
SNHG: small nucleolar RNA host gene
*vIRF1*: viral interferon regulatory factor 1
*HMGB2*: high-mobility group box 2
MALAT1: metastasis-related lung adenocarcinoma transcript 1
EBVaGC: EBV-associated gastric cancer
NEAT1: nuclear paraspeckle assembly transcript 1
HOTAIR: HOX transcript antisense intergenic RNA
EMT: epithelial–mesenchymal transition
VEGFA: vascular endothelial growth factor A.
